# The potential value of quercetin for colorectal cancer: a systematic review and a meta-analysis of preclinical studies

**DOI:** 10.3389/fphar.2025.1642957

**Published:** 2025-09-05

**Authors:** Xiucheng Duan, Liyuan Zhang, Fenye Liu

**Affiliations:** ^1^ The First School of Clinical Medicine, Shandong University of Traditional Chinese Medicine, Jinan, China; ^2^ Shandong Second Provincial General Hospital, Jinan, China; ^3^ Shandong University of Traditional Chinese Medicine, Jinan, China; ^4^ Department of Traditional Chinese Medicine, Shandong Provincial Hospital Affiliated to Shandong First Medical University, Jinan, China

**Keywords:** quercetin, colorectal cancer, animal model, meta analysis, systematic review

## Abstract

**Objective:**

Quercetin, a ubiquitous natural flavonoid present in numerous medicinal plants and foods, has been widely recognized for its various bioactive properties. However, despite its potential, the preclinical animal studies evaluating its therapeutic efficacy in colorectal cancer (CRC) remain inadequate, and the existing clinical research in this area is still limited in quantity. These deficiencies hinder the practical application of quercetin in the treatment of colorectal cancer.

**Methods:**

Our comprehensive review involved systematically searching major databases—including PubMed, Web of Science, and Embase—up to April 2025 for relevant preclinical studies. The SYRCLE risk of bias tool was employed by researchers to evaluate each entry. Subsequently, data analysis was conducted using the statistical software Review Manager 5.4.

**Results:**

The results of our meta-analysis showed that quercetin treatment not only significantly reduced the incidence of CRC (SMD-1.22, 95% CI: −0.26 to −0.38, p = 0.004), but also alleviated inflammation and oxidative stress compared with the control group. Quercetin treatment effectively improved the degree of crypt lesions (SMD-1.40, 95%CI: −2.53 to −0.26, p = 0.02) and alleviated precancerous lesions in the animal model of CRC. In terms of tumor cell proliferation, quercetin had a significant inhibitory effect on cell proliferation during treatment, as determined by PCNA analysis (SMD -8.22, 95% CI: −10.48 to −5.95, p < 0.00001). Quercetin may promote apoptosis during treatment, but this hypothesis has not been supported.

**Conclusion:**

Our study indicates that quercetin exerts beneficial effects across multiple facets of CRC treatment. Nonetheless, precise evaluation of quercetin’s impact on colorectal cancer demands further high-quality, large-scale animal and human studies to confirm our findings.

**Systematic Review Registration:**

https://inplasy.com, identifier INPLASY202550014.

## Graphical Abstract

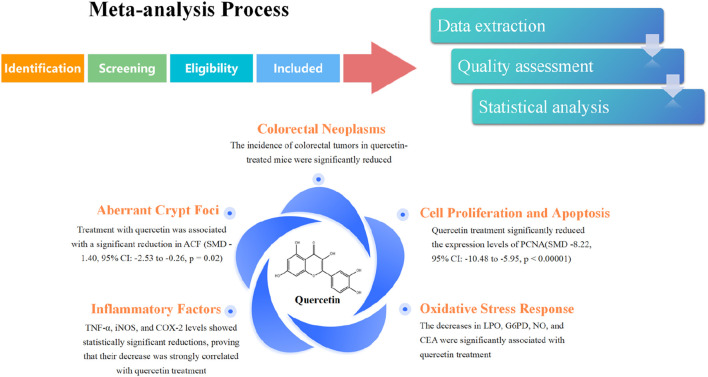

## 1 Introduction

Colorectal cancer ranks as the third most common cancer worldwide, accounting for roughly 10% of all cancer cases (Klimeck et al., 2023). Meanwhile, colorectal cancer claims around 940,000 lives annually, accounting for 9.4% of all cancer-related deaths and placing it as the second most common cause of cancer-related mortality (Roshandel et al., 2024; Hossain et al., 2022). In addition to the limited treatment options available at early stages and the constraints of late-stage targeted therapies, numerous challenges persist in the clinical management of the disease (Chakrabarti et al., 2020; Ciracì et al., 2025). For instance, chemotherapeutic regimens involving substances such as 5-FU exhibit limited clinical efficacy due to the development of drug resistance (Zhang et al., 2008). Off-target effects and drug resistance associated with targeted therapies aimed at VEGF and EGFR are prevalent (Wang et al., 2023; Chong and Jänne, 2013). Chemotherapy and targeted therapies are associated with neurotoxicity, cardiotoxicity, and other detrimental side effects, which significantly impact the patients’ quality of life (Mudd et al., 2021). Thus, identifying new therapeutic agents for colorectal cancer treatment is of pressing importance.

Quercetin, a natural flavonoid ubiquitous in plants, stands out as a key member of dietary polyphenols (Salehi et al., 2020). Quercetin is recognized as the most consumed flavonoid. It possesses a wide array of significant biological activities and is crucial for processes such as anti-inflammation, antioxidation, and anticancer actions (Aghababaei and Hadidi, 2023; Kopustinskiene et al., 2020). However, current animal studies examining quercetin’s efficacy in treating colorectal cancer reveal discrepancies between the results of different experiments. For example, a 2008 study by Cynthia A. Warren showed that quercetin treatment significantly reduced the severity of crypt lesions, an early indicator of colorectal cancer (Warren et al., 2009). This result contradicts the findings of E.E. Deschner in 2013. Moreover, certain animal experiments have shown that the therapeutic effects of quercetin could not be statistically validated (Deschner et al., 1991). Such discrepancies are also observed in other relevant indicators. A 2016 study by Q.C. Li found that quercetin-treated mice exhibited significant body weight loss compared to control mice (Li et al., 2016). This finding contrasts with a 2024 study, which observed significant weight gain in mice treated with quercetin, highlighting a considerable discrepancy between the two studies (Pérez-Valero et al., 2024). Moreover, several animal studies have indicated that the therapeutic effects of quercetin were not statistically significant. Therefore, a statistical analysis of quercetin’s therapeutic effects is warranted.

Currently, animal studies on quercetin’s efficacy in treating colorectal cancer have yielded inconsistent results. To tackle this problem, reduce random error and minimize publication bias, boost statistical power, and reveal patterns that single studies might miss, a meta-analysis becomes necessary (Mathur and Vanderweele, 2021). Therefore, this systematic review and meta-analysis endeavors to conduct a comprehensive evaluation of relevant animal studies, determine the therapeutic efficacy of quercetin on colorectal cancer, investigate its underlying mechanisms, and furnish a scientific foundation for its clinical utilization in colorectal cancer treatment.

## 2 Methods

Our research adheres to the Cochrane Handbook and PRISMA guidelines, and is registered on the International Platform of Registered Systematic Review and Meta-analysis Protocols at https://inplasy.com, with registration number INPLASY202550014 and DOI 10.37766/inplasy2025.5.0014.

### 2.1 Search strategy

In our study, we retrieved relevant studies published in various languages from the inception of the Embase, Web of Science, and PubMed databases up to April 2025. The search employed terms like “Quercetin,” “Isoquercitrin,” “colorectal cancer,” “Colorectal Neoplasms,” “Colitis-Associated Neoplasms,” and “Lynch Syndrome” across all databases, combined with MeSH terms, and used Boolean operators AND and OR. Supplementary studies were found via relevant study reference lists. A comprehensive and systematic retrieval process was implemented, tailored to the specific characteristics of each database. The retrieved literature was imported into EndNote software. The full search protocol is provided in the Supplementary Material.

### 2.2 Study selection

**FIGURE 1:**
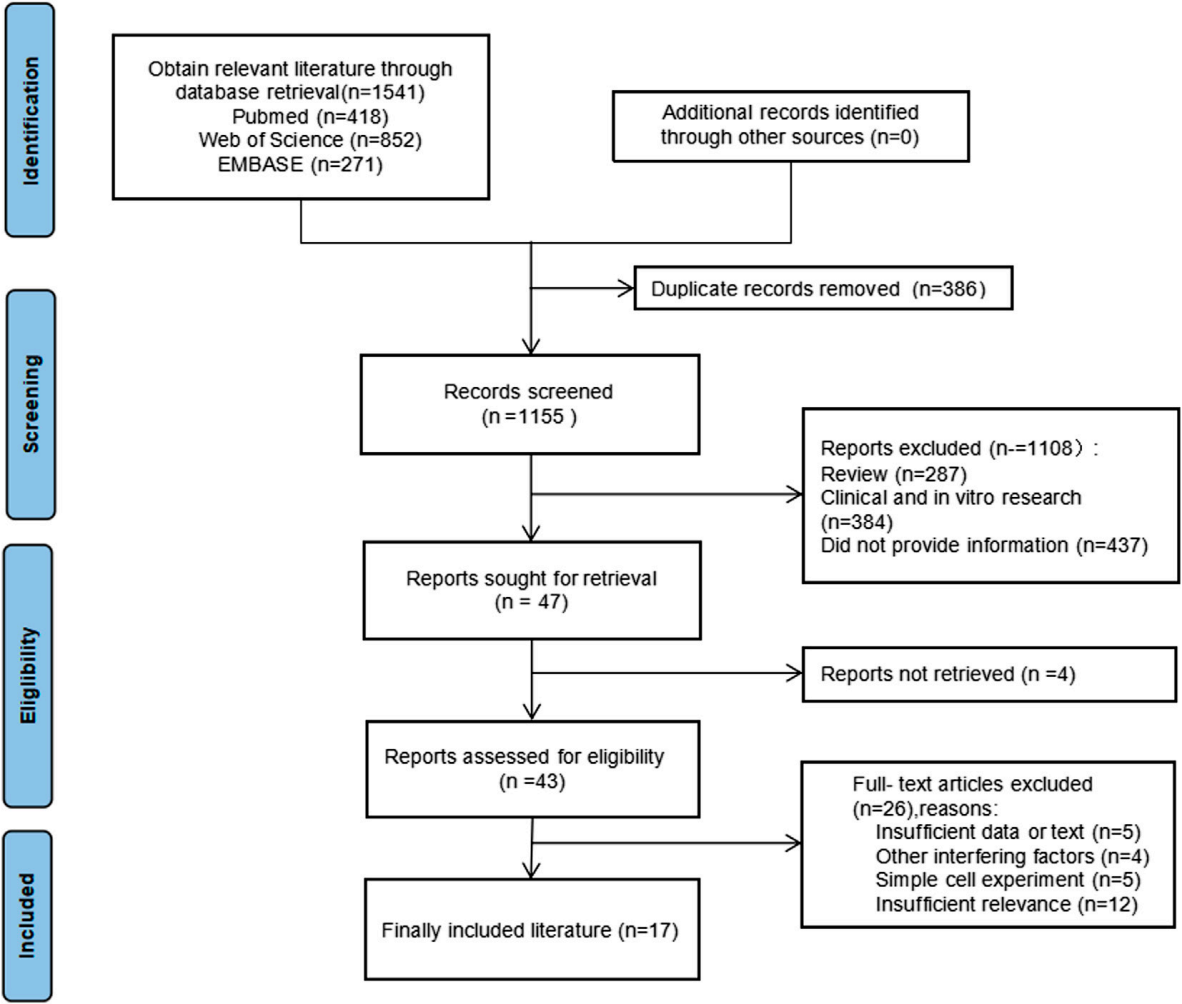
PRISAMA flow chart.

Study eligibility was defined according to the PICOS principles, as recommended by PRISMA: Population (P): healthy rodents capable of inducing colorectal cancer, with an animal model that meets the treatment criteria for colorectal cancer; Intervention (I): Quercetin was administered to the experimental group, whereas the control group remained untreated. The sole difference in the intervention between the two groups was the application of quercetin treatment; Control (C): Colorectal cancer induction was performed accordingly; Outcome (O): Relevant outcome indicators for colorectal cancer in the controlled experiment, including iNOS, COX-2, SOD, GSH, GST, SOD, LPO, CAT, G6PD, CEA, NO, PCNA, BCL-2, IL-2β, ACF, β-catenin, caspase, TNF-α, tumor size, and weight; Research Design (S): Randomized controlled trial. The PRISAMA flow chart is shown in Figure 1.

Exclusion criteria: 1 Non-peer-reviewed sources, including reviews, case reports, guidelines, and conference abstracts; 2 Clinical trials and *in vitro* experiments; 3 Duplicate or off-topic literature; 4 Animal models not specific to colorectal cancer; 5 Studies where drug combinations or other substances might confound results; 6 Documents or data unavailable in full text.

### 2.3 Data extraction

Two researchers (Xiucheng Duan and Liyuan Zhang) independently screened the literature using Endnote software. Any discrepancies were resolved by a third researcher (Fenye Liu). The primary extracted data include: 1 Basic information from the literature, such as the title, name of the first author, publication year, country, animal model information, modeling method, and administration method; 2 Intervention measures, including drug names, dosages, administration times, and frequencies; 3 Outcome measures related to the article. For studies reporting research data in image form, experimental data were extracted using the Engauge Digitizer software.

### 2.4 Quality assessment

Two researchers (Xiucheng Duan and Liyuan Zhang) used the SYRCLE risk of bias tool to assess each entry, making judgments on low-risk, high-risk, and unclear categories based on the appropriate criteria. The standard criteria included sequence generation (selection bias), baseline characteristics (selection bias), allocation concealment (selection bias), random housing (performance bias), blinding (performance bias), random outcome assessment (detection bias), blinding (detection bias), incomplete outcome data (attrition bias), selective outcome reporting (reporting bias), and other sources of bias (other). Any disagreements were resolved through discussion with a third researcher.

### 2.5 Statistical analysis

Data were analyzed using the statistical software Review Manager 5.4. The forest plot was generated using this software. The 95% confidence interval (95% CI) was determined using either the mean difference (MD) or the standardized mean difference (SMD). Given the variability in intervention protocols, such as differences in drug dosage and trial duration, a random effects model was used for the analysis. In our analysis, the I^2^ statistic was employed to assess heterogeneity, with its values interpreted as follows: low (<30%), moderate (30%–60%), substantial (50%–90%), and considerable (75%–100%). Sensitivity analyses, including elimination-by-exclusion, may be necessary in comprehensive meta-analyses due to potential selection bias, small sample sizes, and true heterogeneity. Funnel plots were employed to evaluate publication bias. We established the threshold for statistical significance at a p-value of 0.05. Should the p-value fall below 0.05, it indicates the presence of statistically significant heterogeneity among the studies. For studies with excessive heterogeneity, further analysis should be conducted to identify the source of heterogeneity.

## 3 Results

A total of 1,541 records were screened, and 47 studies were further evaluated according to the modified PRISMA guidelines. After applying the inclusion and exclusion criteria, 17 studies were selected for incorporation into the systematic review and meta-analysis. The experimental information from these studies is detailed in Table 1.

**TABLE 1:** Information about experiments in the literature was included.

Study	Country	Year	Animal characteristics	Model	Control	Inversion	Outcome
Sajjad Tezerji	Iran	2022	Sprague Dawley	Colon cancer	AOM	Quercetin	β-catenin
			n = 45		Length:126 days	Length of intervation:140 days	caspase
			male	material: AOM	Dosages:15 mg/kg bw	Dosages:10 mg/kg bw	Bcl-2
			200–220 g (3–6 weeks)				
Alpa Shree	India	2020	Wistar rats	Colon cancer	DMH	Quercetin	β-catenin
			n = 32		Length:98 days	Length of intervation:105 days	iNOS LPO COX-2
			female	material: DMH	Dosages:20 mg/kg bw	Dosages:50 mg/kg bw	GSH GST
			125–165 g				PCNA Bcl-2
Shirin Sadighparvar	Iran	2020	Wistar rats	Colon cancer	DMH	Quercetin	β-catenin
			n = 30		Length:84 days	Length of intervation:84 days	TNF-α IL-1β
			male	material: DMH	Dosages:20 mg/kg bw	Dosages:20 mg/kg bw	Tumor size
			80–100 g		Freq: once a week for 10 weeks	Freq:1 mL per rat daily	Incidence
Saber Ghazizadeh Darband	Iran	2020	Wistar rats	Colon cancer	DMH	Quercetin	Weight (>15W)
			n = 60		Length:210 days	Length of intervation:210 days	GSH GST
			male	material: DMH	Dosages:20 mg/kg bw	Dosages:50 mg/kg bw	CAT SOD
			80–100 g		Freq: once a week for 15 weeks	Freq:5 days each week	PCNA
TH Saleem	Egypt	2015	albino mice	Colon cancer	DMH	Quercetin	GSH LPO
			n = 60		Length:35 days	Length of intervation:35 days	G6PD CEA
			male	material: DMH	Dosages:25 mg/kg bw	Dosages:50 mg/kg bw	NO CAT
			25–30 g		Freq: once a week for 5 weeks	Freq: daily for 5 weeks	
Cynthia A. Warren	America	2008	Sprague-Dawley rats	Colon cancer	AOM	Quercetin	ACF(AOM)
			n = 40		Length:35 days	Length of intervation:56 days	COX-2 iNOS
			male	material: Diets	Dosages:15 mg/kg bw	Dosages:4.5 g/kg	
					Freq: once a week for 5 weeks	Freq: daily for 8 weeks	
Michael A. Pereira	America	1996	Fischer 344 rats	Colon cancer	AOM	Quercetin	Weight (<15W)
			n = 216		Length:70 days	Length of intervation:70 days	Incidence (AOM)
			male	material: AOM	Dosages:15 mg/kg bw	Dosages:30 g/kg	
					Freq:8 and 15 days	Freq:8 and 15 days	
Eleanor E. Deschner	America	2009	CF1 mice	Colon cancer	AOM	Quercetin	ACF(AOM)
			n = 98		Length:48 days	Length of intervation:48 days	
			female	material: AOM	Dosages:10 mg/kg bw	Dosages:5%	
			5 weeks of age		Freq: once a week for 6 weeks	Freq: 2 weeks	
Eleanor E. Deschner	America	1991	CF1 mice	Colon cancer	AOM	Quercetin	ACF(AOM)
			n = 390		Length:56 days	Length of intervation:56 days	Incidence (AOM)
			female	material: AOM	Dosages:10 mg/kg bw	Dosages:4%	
			5 weeks of age		Freq: once a week for 6 weeks	Freq: 2 weeks	
Álvaro Pérez-Valero	Spain	2024	Fischer 344 rats	Colon cancer	phosphate buffered saline	Quercetin	Weight (>15W)
			n = 40		Length:126 days	Length of intervation:126 days	Tumor number
			male		Dosages:200 µL	Dosages:25 mg/kg bw	
					Freq:3 days a week for 18 weeks	Freq:3 days a week for 18 weeks	
Daniel de Castilho da Silva	Brazil	2021	xeno-transplantation mice	Colon cancer	saline	isoquercetin	Tumor size
			n = 15		Length:7 days	Length of intervation:7 days	Weight (<15W)
				transplantation	Dosages:0.9%	Dosages:0.5 mL	
				
Valeria Tutino	Italy	2018	C57BL/6J mice	Colon cancer	AOM	Quercetin	Bcl-2
			n = 40		Length:42 days	Length of intervation:42 days	
			male	material: AOM	Dosages:10 mg/kg bw	Dosages:0.5%	
			(5 weeks of age)		Freq: once a week for 6 weeks	Freq: once a week for 6 weeks	
Qing-Chun Li	China	2015	SD rats	Colon cancer	DMH	Quercetin	Weight (<15W)
			n = 32		Length:70 days	Length of intervation:70 days	ACF
			male	material: DMH	Dosages:20 mg/kg bw	Dosages:50 mg/kg bw	
			170–190 g		Freq:10 weeks	Freq: daily for 1 week	
Ruilin	China	2020	C57BL/6J mice	Colon cancer	AOM/DSS	Quercetin	Weight (<15W)
			n = 30		Length:42 days	Length of intervation:42 days	ACF
				material: AOM/DSS	Dosages:10 mg/kg AOM+2% DSS (w/v)	Dosages:30 mg/kg bw	GSH LPO SOD CAT G6PD NO
				
Ashwin A. Dihal	Netherlands	2006	F344 rats	Colon cancer	AOM	Quercetin	ACF(AOM)
			n = 210		Length:70 days	Length of intervation:70 days	β-catenin
			male	material: AOM	Dosages:15 mg/kg bw	Dosages:10 g/kg bw	Incidence (AOM)
			40–90 g (4 weeks of age)		Freq: once a week for 8 weeks		
Firli Rahmah Primula DEWI	Indonesia	2023	Wistar rats	Colon cancer	MNU	Quercetin	Weight (<15W)
			n = 24		Length:42 days	Length of intervation:42 days	caspase
			female	material: MNU	Dosages:10 mg/kg bw	Dosages:40 mg/kg bw	CEA
			50–60 g (4–6 weeks of age)		Freq:3 times a week for 4 weeks	Freq: twice a week for 4 weeks	
Changwon Yang	Korean	2022	C57BL/6 mice	Colon cancer	AOM/DSS	Quercetin	TNF-α IL-1β
			n = 120				
			male	material: AOM/DSS	Dosages:10 mg/kg AOM+2.5% DSS	Dosages:30 mg/kg bw	
			(6 weeks of age)			Freq: daily	

Rodents were used in all studies included in this meta-analysis. Nine of the selected studies were published in 2020 or later, ensuring the timeliness of the literature. The studies included more male rodents, and all the rodents had varying body weights. The methods for establishing colorectal cancer models in the control groups varied: seven studies used AOM inducers, five used DMH, two used AOM and DSS, one used MNU, and the remaining two studies used normal saline as a blank control. In the experimental group, both the dose and treatment duration of quercetin were consistent, but the experimental outcome measures varied across different studies. Detailed information on the rodents, quercetin treatments, and related outcome measures across the various animal experiments is summarized in the table.

**FIGURE 2:**
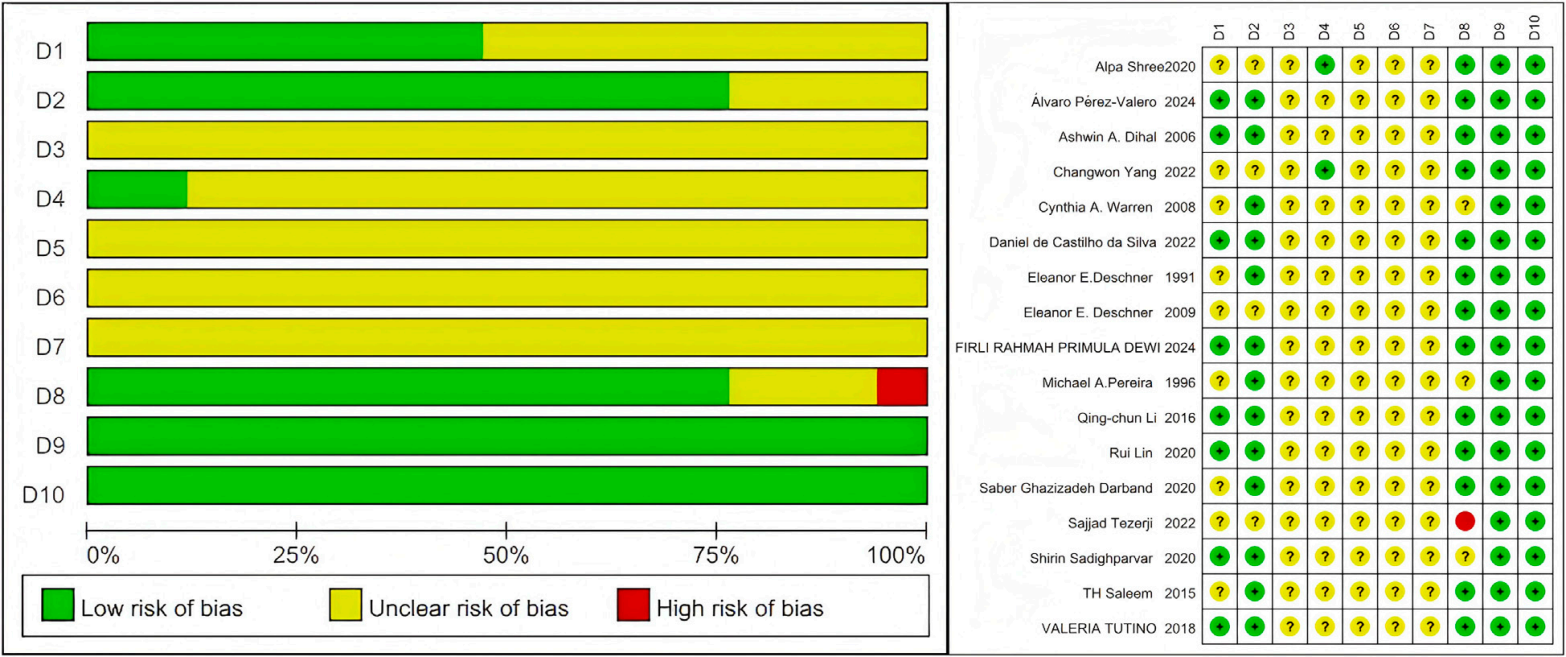
SYRCLE’s risk of bias assessment for included studies.

An assessment of publication bias was carried out on the 17 included studies. The results showed that only one study was identified as having a serious risk of bias due to incomplete data reporting, while the majority of the studies exhibited a low or unclear risk of bias. The bias analysis of most studies focused on 4-5 points, particularly the absence of content related to D3-D7. The results of the specific bias analyses are shown in Figure 2. D1:Sequence generation; D2:Baseline characteristics; D3:Concealed grouping; D4:Randomization of animals’ placement; D5:Blinding of methods during the intervention; D6:Randomisation outcome assessment; D7:Blinding of outcome evaluations; D8:Reporting of incomplete data; D9:Selective data reporting; D10:Other bias analyses.

Methodological limitations were frequently observed in the included studies, particularly regarding the concealment of allocation sequences and the application of blinding. Inadequate reporting or implementation of allocation sequence concealment was common, potentially leading to undetected baseline imbalance between groups. Challenges in applying blinding to participants, intervention implementers, and outcome assessors were also noted, with a lack of blinding, especially among outcome assessors, possibly compromising the objectivity of subjective outcome measurements and potentially causing an overestimation or underestimation of intervention effects.

Given the prevalence of concealment bias and lack of blinding in the included studies, caution is warranted when interpreting the pooled effect sizes from this meta-analysis. These methodological shortcomings may undermine confidence in the internal validity of the results, suggesting that the observed effects might not fully reflect the true intervention effects.

Although implementing blinding can be challenging in certain intervention types, the insufficiency of allocation concealment and the absence of blinding, particularly concerning outcome assessors, represent significant limitations in the studies included in this review. These factors must be carefully considered when interpreting the review’s findings.

## 4 Meta-analysis results (random effects analysis)

### 4.1 Body weight of rodents

Seven studies reported the final body weight of rodents, with 77 rodents in the quercetin treatment group and 76 in the control group. The relationship between quercetin treatment and changes in body weight in rodents remains unclear (SMD 1.08, 95% CI: −0.05 to 2.20, p = 0.06). However, there was no statistically significant heterogeneity between studies, and the outcomes were not statistically significant (Chi^2^ = 44.90, p < 0.00001, I^2^ = 87%).

Subgroup analyses were performed according to trial duration. In two studies exceeding 15 weeks, 18 rodents were assigned to quercetin and 18 to control; a strong association between quercetin and body-weight change was observed (SMD 0.75, 95% CI 0.07 to 1.44, p = 0.03), and no significant heterogeneity was detected across studies (Chi^2^ = 0.34, p = 0.56, I^2^ = 0%).

**FIGURE 3:**
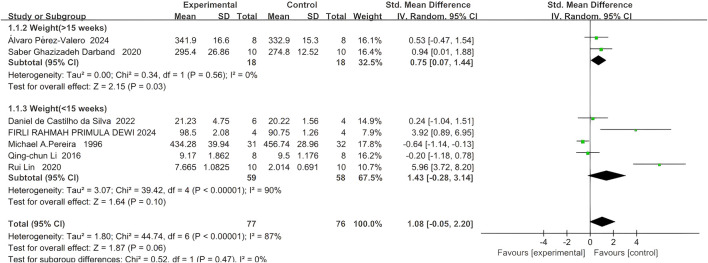
Forest plot for the effects of Quercetin on the Final body weight of Rodents compared with the model group (Conducted subgroup analysis based on the Research duration).

In five trials shorter than 5 weeks, 59 rodents received quercetin and 58 served as controls; here, a strong association between treatment and body-weight change was similarly noted (SMD 1.43, 95% CI –0.28 to 3.14, p = 0.10), whereas significant heterogeneity was present (Chi^2^ = 39.42, p < 0.00001, I^2^ = 90%). The specific data are shown in Figure 3.

### 4.2 Colorectal neoplasms

#### 4.2.1 Incidence of colorectal neoplasms

Five studies documented the incidence of colorectal cancer, comprising 103 rodents in the quercetin group and 106 in the control group. Quercetin administration was linked to a substantial decrease in colorectal cancer incidence (SMD −1.22, 95% CI: −2.06 to −0.38, p = 0.004). Statistically significant heterogeneity was observed across the studies (Chi^2^ = 21.81, p = 0.0002, I^2^ = 82%).

Subgroup analyses were conducted based on the colorectal carcinogenesis protocols. Three studies used azoxymethane (AOM) induction. In these experiments, quercetin was administered to 92 rodents and 90 received vehicle. A strong inverse association between quercetin exposure and tumor incidence was demonstrated (SMD −0.58, 95% CI: −0.87 to −0.28, p = 0.0001). No significant heterogeneity was found between the studies (Chi^2^ = 0.95, p = 0.62, I^2^ = 0%).

**FIGURE 4:**
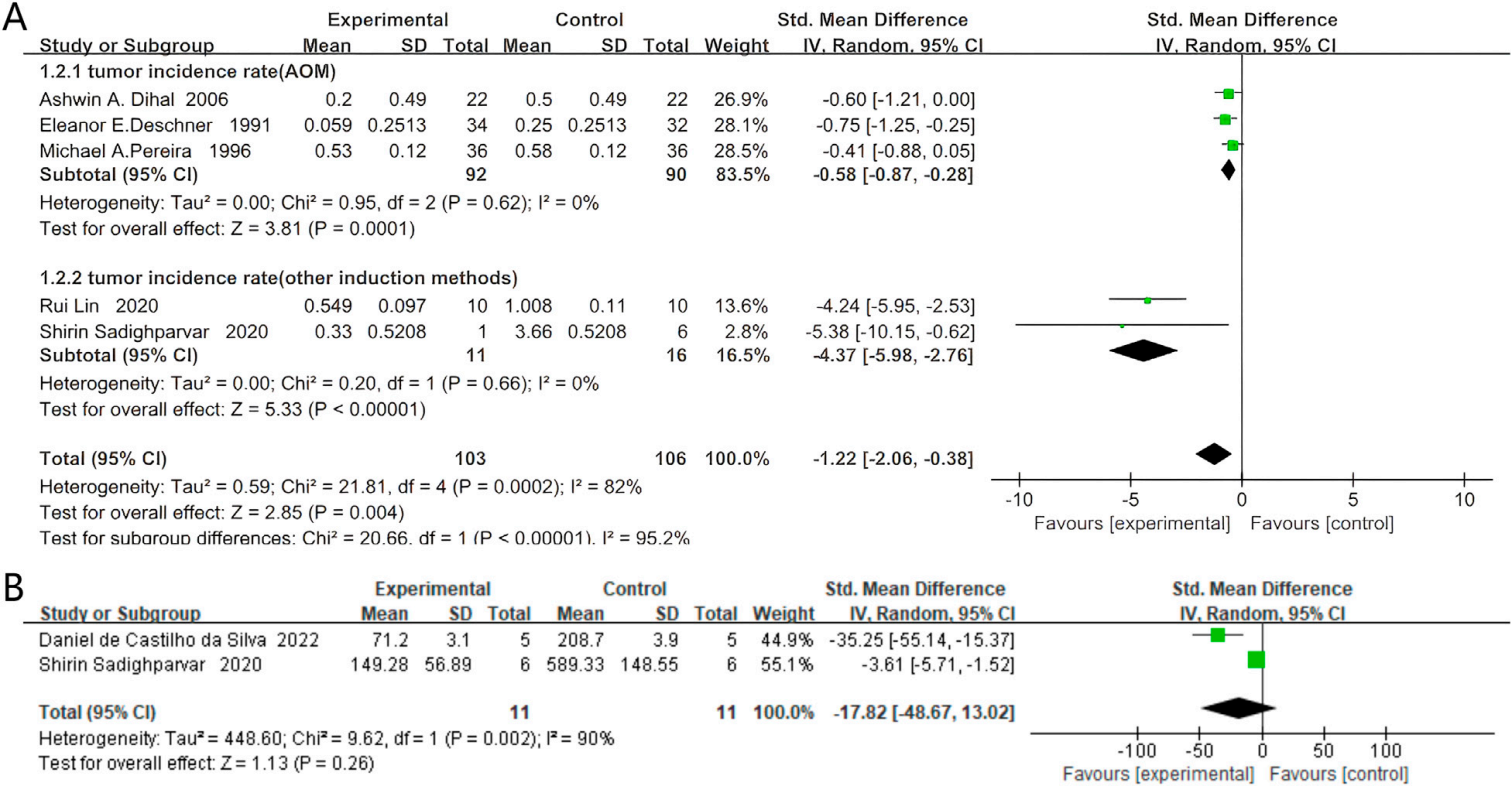
Forest plot for the effects of Quercetin on the Colorectal neoplasms of Rodents compared with the model group. **(A)** Forest plot for the effects of Quercetin on the incidence of colorectal neoplasms compared with the model group (Conducted subgroup analysis based on the induction method). **(B)** Forest plot for the effects of Quercetin on the tumor size of colorectal tumors compared with the model group.

Two studies employed alternative induction methods. Here, 11 rodents were allocated to quercetin and 16 to control. A strong inverse relationship between quercetin treatment and tumor incidence in long-term rodent models was revealed (SMD −4.37, 95% CI: −5.98 to −2.76, p < 0.00001). No evidence of statistical heterogeneity was observed between these studies (Chi^2^ = 0.20, p = 0.66, I^2^ = 0%). The specific data are shown in Figure 4A.

#### 4.2.2 Tumor size of colorectal tumors

Two studies documented the tumor size of colorectal tumors, with 11 rodents in the quercetin group and 11 in the control group. Quercetin administration was linked to a potential reduction in tumor size (SMD −17.82, 95% CI: −48.67 to 13.02, p = 0.26). However, the heterogeneity between studies was not found to be statistically significant, and the results did not reach statistical significance (Chi^2^ = 9.62, p = 0.002, I^2^ = 90%). The specific data are presented in Figure 4B.

### 4.3 Aberrant crypt foci (ACF)

Six studies reported ACF, with 76 rodents in the quercetin treatment group and 76 in the control group. Treatment with quercetin was associated with a significant reduction in ACF (SMD −2.05, 95% CI: −3.21 to −0.90, p = 0.0005). Statistically significant heterogeneity was observed across the studies (Chi^2^ = 30.55, p < 0.0001, I^2^ = 85%).

Further subgroup analyses of aberrant crypt foci (ACF) were stratified by colorectal carcinogenesis protocol. In four azoxymethane (AOM)-based studies, 60 rodents received quercetin and 60 received vehicle; a strong inverse association between quercetin exposure and ACF count was observed (SMD –1.47, 95% CI –2.77 to −0.17, p = 0.03), whereas significant heterogeneity was detected across studies (Chi^2^ = 21.98, p < 0.0001, I^2^ = 86%).

**FIGURE 5:**
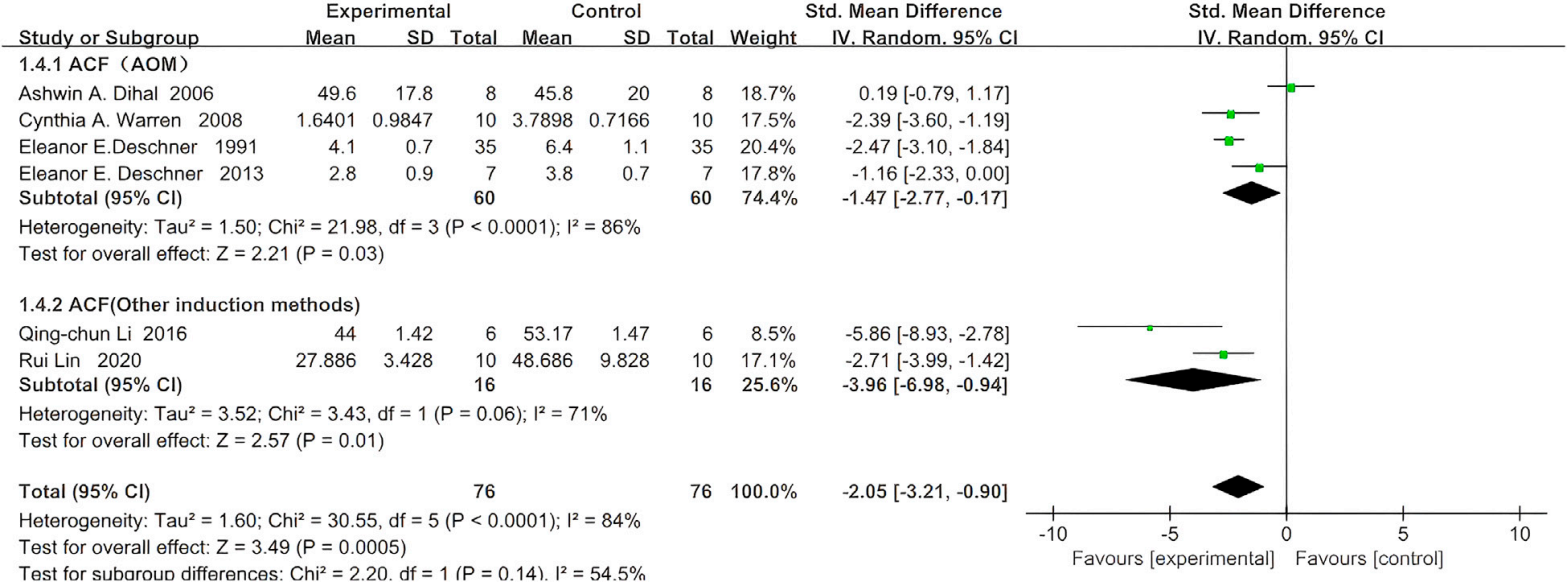
Forest plot for the effects of Quercetin on Aberrant Crypt Foci compared with the model group (Conducted subgroup analysis based on the induction method).

In two studies employing alternative induction methods, 16 rodents were allocated to quercetin and 16 to control; a similarly strong inverse relationship was demonstrated (SMD –3.96, 95% CI –6.98 to −0.94, p = 0.01), again with significant heterogeneity (Chi^2^ = 3.43, p = 0.06, I^2^ = 71%). The specific data are shown in Figure 5.

### 4.4 Related indicators of cell proliferation and apoptosis

#### 4.4.1 Caspase

**FIGURE 6:**
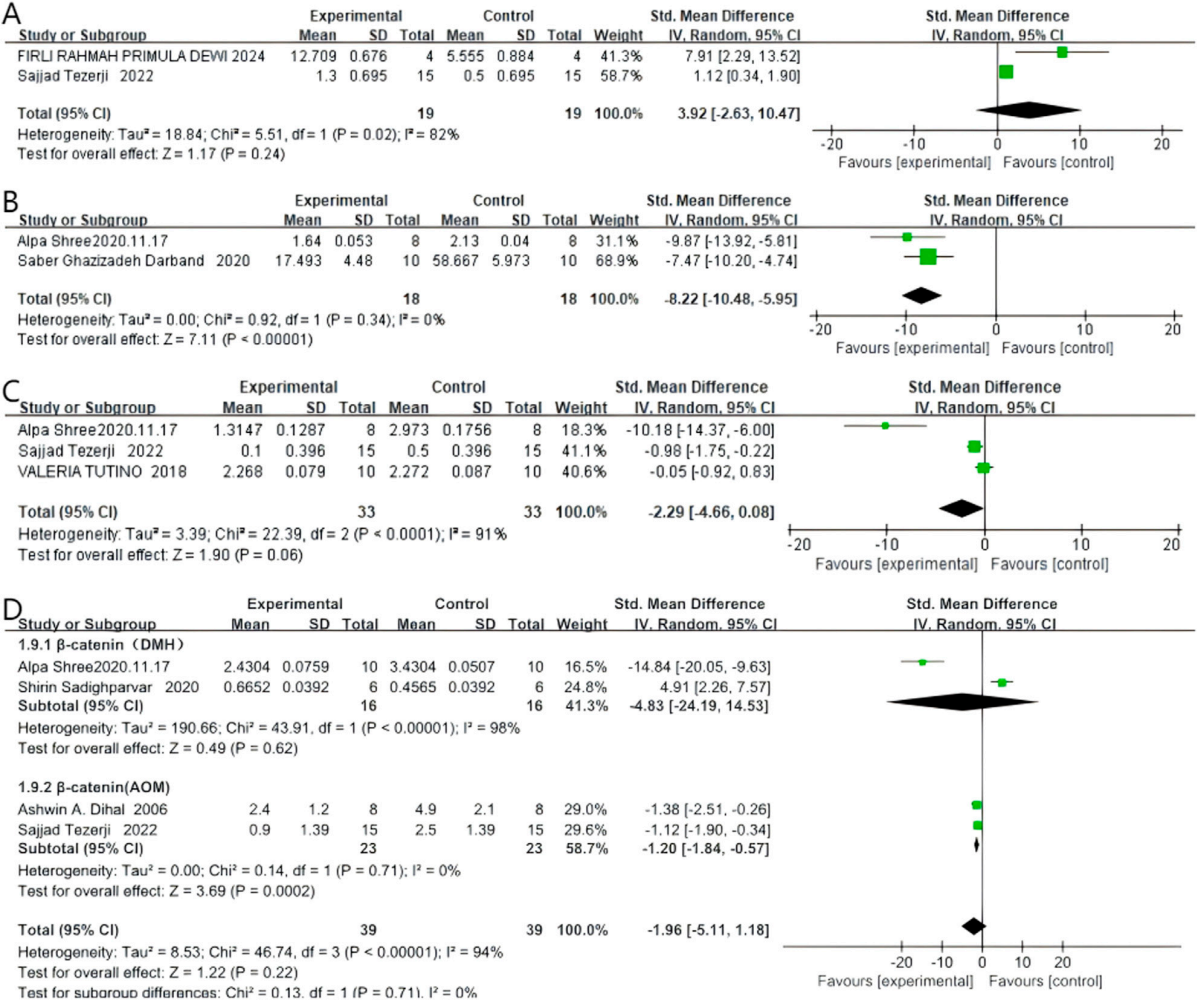
Forest plot for the effects of Quercetin on Related indicators of cell proliferation and apoptosis compared with the model group. **(A)** Forest plot for the effects of Quercetin on Caspase with the model group. **(B)** Forest plot for the effects of Quercetin on PCNA with the model group. **(C)** Forest plot for the effects of Quercetin on Bcl-2 with the model group. **(D)** Forest plot for the effects of Quercetin on β-Catenin with the model group (Conducted subgroup analysis based on the induction method).

Two studies reported Caspase levels, with 19 rodents in the quercetin treatment group and 19 in the control group. Quercetin treatment may be associated with increased Caspase levels (SMD 3.92, 95% CI: −2.63 to 10.47, p = 0.24). However, no statistically significant heterogeneity was observed between studies, and the outcomes were not statistically significant (Chi^2^ = 5.51, p = 0.02, I^2^ = 82%). Therefore, the effect of quercetin on Caspase levels during CRC treatment remains unclear and requires further investigation. The specific data are shown in the Figure 6A.

#### 4.4.2 Proliferating cell nuclear antigen (PCNA)

Two studies reported PCNA expression, with 18 rodents in the quercetin treatment group and 18 in the control group. Quercetin treatment significantly reduced PCNA expression levels (SMD −8.22, 95% CI: −10.48 to −5.95, p < 0.00001). No statistically significant heterogeneity was detected among the studies (Chi^2^ = 0.92, p = 0.34, I^2^ = 0%). Thus, quercetin inhibits proliferation markers in colorectal cancer. The specific data are presented in Figure 6B.

#### 4.4.3 B-cell lymphoma-2 (Bcl-2)

Three studies reported Bcl-2 expression, with 33 rodents in the quercetin treatment group and 33 in the control group. Quercetin treatment may be associated with a reduction in Bcl-2 expression levels (SMD −2.29, 95% CI: −4.66 to 0.08, p = 0.06). However, the heterogeneity between studies was not statistically significant, and the results did not reach statistical significance (Chi^2^ = 22.39, p < 0.0001, I^2^ = 91%). Therefore, the effect of quercetin on Bcl-2 expression during CRC treatment remains unclear and requires further investigation. The specific data are shown in the Figure 6C.

#### 4.4.4 β-Catenin

Four studies reported β-Catenin expression, with 39 rodents in the quercetin treatment group and 39 in the control group. Quercetin treatment may be associated with a reduction in β-Catenin expression levels (SMD −1.96, 95% CI: −5.11 to 1.18, p = 0.22). However, no statistically significant heterogeneity was observed between studies, and the outcomes were not statistically significant (Chi^2^ = 46.74, p < 0.00001, I^2^ = 94%). Therefore, the effect of quercetin on β-Catenin expression during CRC treatment remains unclear and requires further investigation. The specific data are shown in the Figure 6D.

### 4.5 Expression of inflammatory factors

#### 4.5.1 TNF-α

**FIGURE 7:**
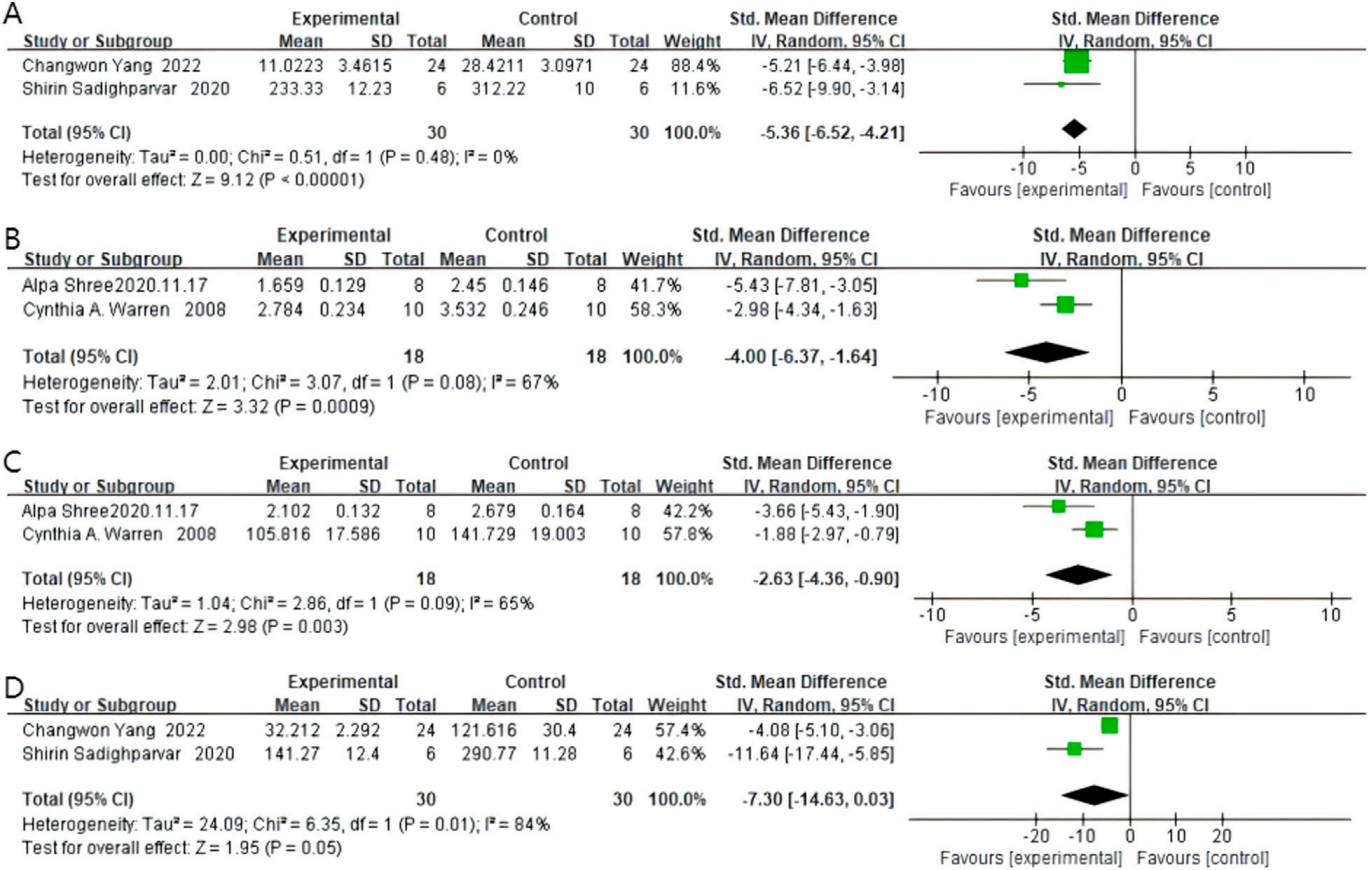
Forest plot for the effects of Quercetin on inflammatory factors compared with the model group. **(A)** Forest plot for the effects of Quercetin on TNF- compared with the model group. **(B)** Forest plot for the effects of Quercetin on iNOS compared with the model group. **(C)** Forest plot for the effects of Quercetin on COX-2 compared with the model group. **(D)** Forest plot for the effects of Quercetin on IL-1 compared with the model group.

Two studies reported TNF-α expression, with 30 rodents in the quercetin treatment group and 30 in the control group. Quercetin treatment significantly reduced TNF-α expression levels (SMD −5.36, 95% CI: −6.52 to −4.21, p < 0.00001). No statistically significant heterogeneity was observed across the studies (Chi^2^ = 0.51, p = 0.48, I^2^ = 0%). Therefore, TNF-α expression was significantly inhibited by quercetin. The specific data are shown in Figure 7A.

#### 4.5.2 iNOS

Two studies reported iNOS levels, with 18 rodents in the quercetin treatment group and 18 in the control group. Quercetin treatment significantly reduced iNOS levels (SMD −4.00, 95% CI: −6.37 to −1.64, p = 0.0009). Statistically significant heterogeneity was observed across the studies (Chi^2^ = 3.07, p = 0.08, I^2^ = 67%). Therefore, quercetin has an anti-inflammatory effect in colorectal cancer. The specific data are shown in Figure 7B.

#### 4.5.3 COX-2

Two studies reported COX-2 levels, with 18 rodents in the quercetin treatment group and 18 in the control group. Quercetin treatment significantly reduced COX-2 levels (SMD −2.63, 95% CI: −4.36 to −0.90, p = 0.003). Statistically significant heterogeneity was observed across the studies (Chi^2^ = 2.86, p = 0.09, I^2^ = 65%). The specific data are shown in Figure 7C.

#### 4.5.4 IL-1β

Two studies reported IL-1β levels, with 30 rodents in the quercetin treatment group and 30 in the control group. Quercetin treatment significantly reduced IL-1β levels (SMD −7.30, 95% CI: −14.63 to 0.03, p = 0.05). However, the heterogeneity between studies was not statistically significant, and the outcomes did not reach statistical significance either (Chi^2^ = 6.35, p = 0.01, I^2^ = 84%). The specific data are shown in Figure 7D.

### 4.6 Oxidative stress response

#### 4.6.1 GSH

Four studies reported GSH levels, with 40 rodents in the quercetin treatment group and 40 in the control group. The relationship between quercetin treatment and changes in GSH levels remains unclear (SMD 0.59, 95% CI: −3.36 to 4.53, p = 0.77). However, no statistically significant heterogeneity was observed between studies, and the outcomes were not statistically significant (Chi^2^ = 78.20, p < 0.00001, I^2^ = 96%). The impact of quercetin on GSH levels during CRC treatment is still unclear and more studies are needed to clarify this relationship.

**FIGURE 8:**
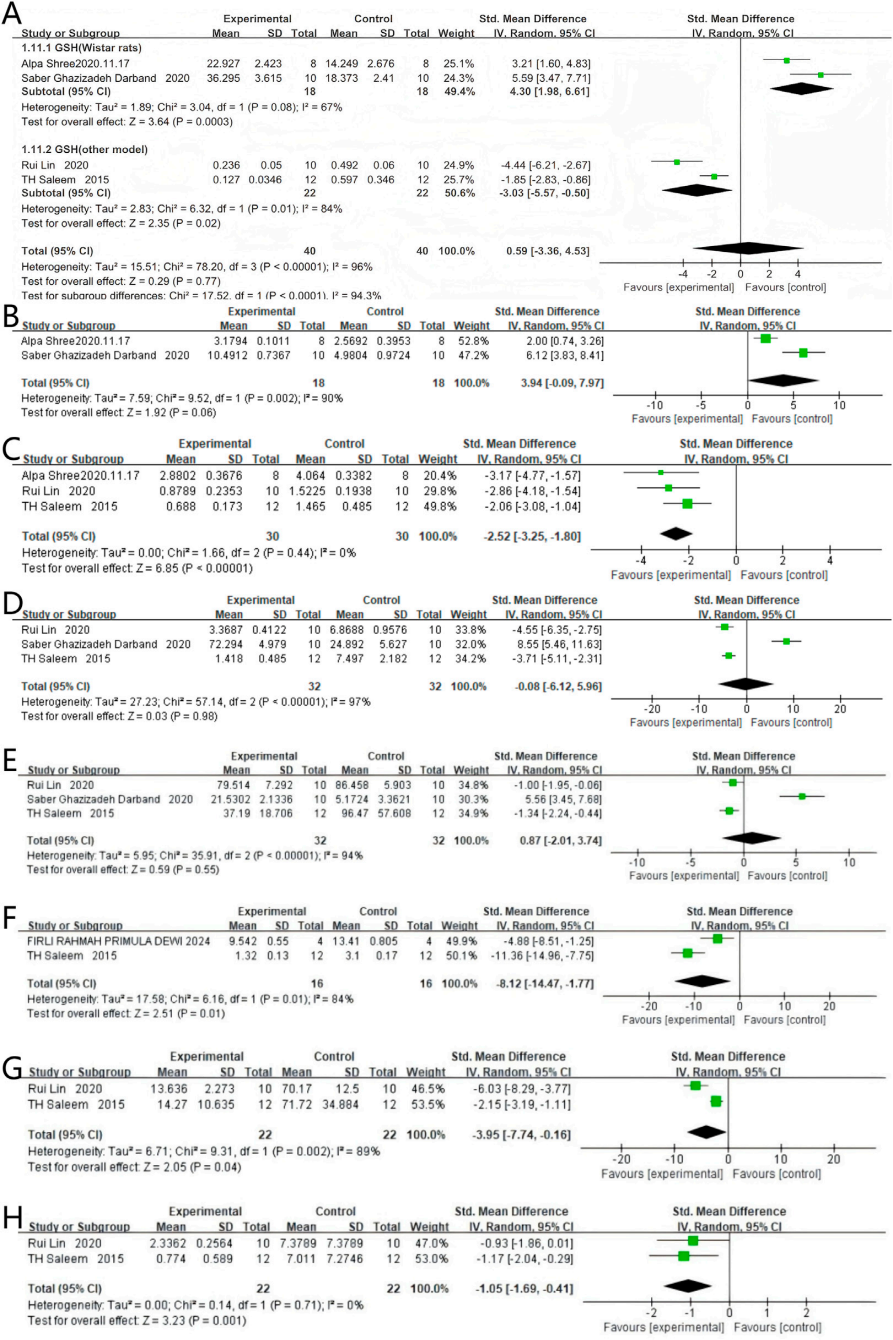
Forest plot for the effects of Quercetin on Oxidative stress response compared with the model group. **(A)** Forest plot for the effects of Quercetin on GSH compared with the model group (Conducted subgroup analysis based on the species of animals). **(B–H)** Forest plots for the effects of Quercetin on GST, LPO, SOD, CAT, CEA, G6PD, and NO compared with the model group.

Subgroup analyses for GSH were stratified by rodent strain. In two Wistar-based studies, quercetin was administered to 18 rats and vehicle to 18 controls; a strong positive association with GSH content was observed (SMD 4.30, 95% CI 1.98–6.61, p = 0.0003), accompanied by significant heterogeneity (Chi^2^ = 3.04, p = 0.08, I^2^ = 67%). In two studies employing other strains, 40 rodents received quercetin and 40 received control; a strong inverse relationship was likewise demonstrated (SMD –0.59, 95% CI –5.57 to −0.50, p = 0.02), with significant heterogeneity (Chi^2^ = 6.32, p = 0.01, I^2^ = 84%). The specific data can be seen in Figure 8A.

#### 4.6.2 GST

Two studies reported GST levels, with 18 rodents in the quercetin treatment group and 18 in the control group. Quercetin treatment may be associated with a significant increase in GST levels (SMD 3.94, 95% CI: −0.09 to 7.97, p = 0.06). However, the outcomes were not statistically significant (Chi^2^ = 9.52, p = 0.002, I^2^ = 90%). Therefore, the effect of quercetin on GST levels during CRC treatment cannot be fully determined and requires further investigation. The specific data are shown in Figure 8B.

#### 4.6.3 LPO

Three studies reported LPO levels, with 30 rodents in the quercetin treatment group and 30 in the control group. Quercetin treatment significantly reduced LPO levels (SMD −2.52, 95% CI: −3.25 to −1.80, p < 0.00001). Statistically significant heterogeneity was not observed across the studies (Chi^2^ = 1.66, p = 0.44, I^2^ = 0%). Therefore, quercetin reduces LPO levels during colorectal cancer treatment. The specific data are shown in Figure 8C.

#### 4.6.4 SOD

Three studies reported SOD levels, with 32 rodents in the quercetin treatment group and 32 in the control group. The relationship between quercetin treatment and changes in SOD levels remains unclear (SMD −0.08, 95% CI: −6.12 to 5.96, p = 0.98). No statistically significant heterogeneity was noted between studies, and the results showed no statistical significance (Chi^2^ = 57.14, p < 0.00001, I^2^ = 97%). The specific data are shown in Figure 8D.

#### 4.6.5 CAT

Three studies reported CAT levels, with 32 rodents in the quercetin treatment group and 32 in the control group. The relationship between quercetin treatment and changes in CAT levels remains unclear (SMD 0.87, 95% CI: −2.01 to 3.74, p = 0.55). However, no statistically significant heterogeneity was observed between studies, and the outcomes were not statistically significant (Chi^2^ = 35.91, p < 0.00001, I^2^ = 94%). Just like SOD, the effect of quercetin on CAT levels during CRC treatment is still not clear and needs more research to understand this relationship. The specific data are shown in Figure 8E.

#### 4.6.6 CEA

Two studies reported CEA levels, with 16 rodents in the quercetin treatment group and 16 in the control group. Quercetin treatment significantly reduced CEA levels (SMD −8.12, 95% CI: −14.47 to −1.77, p = 0.01). Statistically significant heterogeneity was observed across the studies (Chi^2^ = 6.16, p = 0.01, I^2^ = 84%). Therefore, quercetin reduces CEA levels during colorectal cancer treatment. The specific data are shown in Figure 8F.

#### 4.6.7 G6PD

Two studies reported G6PD levels, with 22 rodents in the quercetin treatment group and 22 in the control group. Quercetin treatment significantly reduced G6PD levels (SMD −3.95, 95% CI: −7.74 to −0.16, p = 0.04). The studies showed statistically significant heterogeneity (Chi^2^ = 9.31, p = 0.002, I^2^ = 89%). Therefore, quercetin reduces G6PD levels during colorectal cancer treatment. The specific data are shown in Figure 8G.

#### 4.6.8 NO

Two studies reported NO levels, with 22 rodents in the quercetin treatment group and 22 in the control group. Quercetin treatment significantly reduced NO levels (SMD −1.05, 95% CI: −1.69 to −0.41, p = 0.001). No statistically significant heterogeneity was observed across the studies (Chi^2^ = 0.14, p = 0.71, I^2^ = 0%). Therefore, quercetin reduces NO levels during colorectal cancer treatment. The specific data are shown in Figure 8H.

## 5 Discussion

### 5.1 Data results of the meta-analysis

The meta-analysis results showed that, in terms of mouse signs, the incidence of colorectal tumors and the number of crypt lesions in quercetin-treated mice were significantly reduced, with statistically significant animal experimental data. However, in terms of physical signs, the final body weight of rodents and tumor volume *in vivo* were not statistically significant, and the exact relationship between quercetin treatment and changes in these indicators could not be established. At the cellular level, PCNA, caspase, β-catenin, and Bcl-2 proteins are key factors in regulating cell proliferation and apoptosis (Witko-Sarsat et al., 2010; Parrish et al., 2013; Mcilwain et al., 2013; Olmeda et al., 2003). However, we only confirmed that quercetin treatment is linked to lower PCNA levels, as the other three studies’ results were not statistically significant. Inflammatory mediators like TNF-α, iNOS, COX-2, and IL-1β are central to the inflammatory response (Tajdari et al., 2024). This analysis found that the expression levels of TNF-α, iNOS, and COX-2 were significantly reduced, with statistically significant differences. Although IL-1β showed a decreasing trend, the experimental data were not statistically significant. Finally, regarding indicators related to oxidative stress, the decreases in LPO, G6PD, NO, and CEA were significantly associated with quercetin treatment, showing statistical significance. The remaining four indicators—GAH, GAT, SOD, and CAT—were not statistically significant.

#### 5.1.1 Changes in physical signs of rodents

The effect of quercetin treatment on the final body weight of rodents was unclear, and the data were not statistically significant. This result may be due to various factors, including different rodent breeds (Hundsberger et al., 2021), varying sensitivity to quercetin (Jafarinia et al., 2020), and the effects of different culture cycles on the body. Regarding tumor size in rodents, data analysis showed that the effect of quercetin treatment was inconclusive and not statistically significant, meaning quercetin’s effect on tumor size could not be established. However, regarding cancer incidence, several studies have shown significant reductions with quercetin treatment. Although the effect of quercetin on tumor size remains uncertain, it significantly reduced tumor frequency.

In terms of histological examination in rodents, abnormal crypt lesions are precancerous lesions of colorectal cancer (Roncucci et al., 2000), linked to abnormal Wnt/β-catenin signaling pathways, and serve as risk indicators (Zhao et al., 2022). ACFs can transform into adenomas, which can further develop into colorectal cancer, or directly progress from ACFs to cancer (Petrova et al., 2008). Thus, a higher number of ACFs is linked to a greater risk of colorectal cancer (Kowalczyk et al., 2022). The meta-analysis indicated a significant correlation between quercetin treatment and a reduction in ACFS in rodents. Quercetin treatment may effectively reduce the incidence of precancerous lesions in colorectal cancer, thereby lowering the risk of developing the disease.

#### 5.1.2 Cell proliferation and apoptosis control

According to the meta-analysis, although all four substances were related to changes in the Wnt signaling pathway (Trejo-Solis et al., 2021), only PCNA changes were strongly correlated with quercetin treatment. The other three substances (β-catenin, Bcl-2, and caspase) did not show a strong correlation with quercetin treatment. Additionally, in the β-catenin data extraction, the results from Shirin Sadighparvar’s 2020 study significantly differed from those of other authors in different periods, but the reason for this discrepancy remains unclear (Sadighparvar et al., 2020). Abnormal activation of the Wnt/β-catenin signaling pathway is a hallmark of colorectal cancer development and is closely associated with the proliferation and apoptosis of colorectal tumor cells (Zhao et al., 2022; He and Gan, 2023). Dysregulation of the Wnt signaling pathway can result in mutations or abnormal accumulation of the typically stable β-catenin gene (Maurice and Angers, 2025). On one hand, β-catenin activates downstream target genes, like c-Myc and cyclin D1, thus triggering abnormal cell proliferation (Zhang and Wang, 2020). As a result, PCNA levels, a key protein in DNA replication and a marker of cell proliferation, were significantly increased (Kubben et al., 1994). On the other hand, β-catenin inhibits Bcl-2 levels (Trejo-Solis et al., 2021), which in turn prevents caspase activation, the executor of apoptosis, thereby inhibiting cell death (Trejo-Solis et al., 2021). The meta-analysis results showed that quercetin treatment was strongly correlated with a decrease in PCNA levels, suggesting that quercetin has a significant inhibitory effect on cell proliferation during colorectal cancer treatment. Although the other three measures did not reach statistical significance, the data showed a trend toward decreased β-catenin and Bcl-2 levels, and increased caspase levels. Therefore, quercetin may also promote cell apoptosis during treatment, but this hypothesis is not supported by statistical data and requires further experimental investigation.

#### 5.1.3 Inflammatory factors

The data analysis indicated that quercetin treatment markedly diminished the levels of TNF-α, iNOS, COX-2, and IL-1β, which are all linked to inflammation. Among the four factors, TNF-α, iNOS, and COX-2 levels showed statistically significant reductions, proving that their decrease was strongly correlated with quercetin treatment (Sato and Mukai, 2020). Chronic inflammation is a major driver of colorectal cancer. Inflammatory factors promote carcinogenesis by modulating the tumor microenvironment, inducing gene mutations, and suppressing immune surveillance (Long et al., 2017; Zhang and Qiao, 2022; Percario et al., 2021). During inflammation, macrophages and tumor-associated cells secrete TNF-α and IL-1β, activating the NF-κB pathway and inducing COX-2 expression (Laflamme et al., 1999). iNOS activation is closely related to the COX-2 signaling pathway and is the key enzyme catalyzing NO production (Salvemini et al., 2013; Cinelli et al., 2020). In this process, IL-1β and TNF-α promote M2-type polarization of macrophages and activate angiogenic factors (Pan et al., 2020; Wang et al., 2024; Pérez and Rius-Pérez, 2022), creating a positive feedback loop that exacerbates inflammation and promotes tumor cell proliferation (Gao et al., 2022). Data analysis indicated that quercetin may mitigate the inflammatory impact in colorectal cancer (CRC) treatment by inhibiting the formation and expression of TNF-α. Thus, quercetin’s potential to enhance CRC therapeutic efficacy via suppression of TNF-α expression was highlighted. This line of evidence suggests that quercetin could provide insights into novel therapeutic strategies targeting inflammatory factors. However, whether quercetin can inhibit COX-2 and iNOS expression by reducing IL-1β levels still requires further experimental validation.

#### 5.1.4 Oxidative stress response

GSH, GST, SOD, and CAT are key intracellular antioxidant enzymes in the antioxidant system (Birben et al., 2012). However, the data analysis found no statistically significant differences in the mean values of GSH, SOD, and CAT between the quercetin treatment group and the control group, making it impossible to establish a correlation between quercetin treatment and changes in these markers. So, the function of quercetin in the antioxidant system is still not clear and more experimental data is needed to analyze it. And though the quercetin group had a bit higher GST levels than the control group, the difference was not statistically significant.

In terms of oxidative stress markers, the levels of LPO and NO were significantly decreased in the quercetin treatment group, with both substances linked to tumor invasion and metastasis (Li and Li, 2020). Low concentrations of NO have anti-tumor effects, while high concentrations promote carcinogenesis (Hu et al., 2020). The decrease in NO levels in the quercetin treatment group may be related to quercetin’s inhibitory effect on iNOS. Quercetin may inhibit NO production by reducing iNOS levels or act directly on NO. LPO reflects the extent of oxidative damage to cell membrane lipids, with increased LPO levels typically indicating cellular oxidative stress (Ito et al., 2019). Analysis of quercetin’s effects on oxidative stress markers showed that LPO levels were significantly reduced in the treatment group. This finding suggests quercetin may hold potential as a therapeutic agent targeting LPO in colorectal cancer treatment, as it might lower the invasiveness of tumor cells. However, additional experimental validation is necessary to fully clarify its therapeutic efficacy. G6PD generates NADPH via the pentose phosphate pathway (PPP) to maintain intracellular GSH levels, thereby scavenging ROS (Yang et al., 2021; Yang et al., 2019). Therefore, high G6PD expression not only promotes tumor proliferation and growth but also regulates ROS levels to enhance tumor cell survival (Zhang et al., 2017; Lan et al., 2024). The study showed a strong correlation between G6PD reduction and quercetin treatment, with quercetin significantly reducing G6PD activity. Finally, although CEA itself is not directly involved in oxidative stress, its expression is linked to oxidative stress in the tumor microenvironment and the aforementioned inflammatory response, reflecting the presence and recurrence of tumors (Aboelella et al., 2021). In the data analysis, CEA levels were strongly and statistically significantly associated with quercetin treatment. Given its effects on multiple biological pathways, quercetin may have a unique role in enhancing the efficacy of cancer treatments. It has the potential to be one of the therapeutic options that can stabilize the disease and prevent further progression. However, more research is needed to confirm these effects.

#### 5.1.5 Inference of interactions among key indicators

Quercetin exhibits significant inhibitory effects on multiple key biomarkers, including PCNA, TNF-α, iNOS, COX-2, LPO, G6PD, NO, and CEA. These markers are interconnected through an inflammation-oxidative stress feedback loop: Elevated TNF-α activates NF-κB signaling, subsequently upregulating COX-2 and iNOS expression. Increased iNOS catalyzes excessive NO production, which promotes oxidative damage as evidenced by elevated LPO. Oxidative stress reciprocally reinforces NF-κB activation, thereby amplifying inflammatory responses. In quercetin-treated models, consistent suppression of TNF-α, COX-2, iNOS, NO, and LPO was observed, suggesting potential disruption of this pathogenic cycle.

The reduction in ACF and CEA levels further supports quercetin’s integrated anti-inflammatory, antioxidant, and antiproliferative efficacy. Notably, unchanged levels of antioxidant enzymes (GSH, SOD, CAT) indicate that oxidative stress amelioration may be mediated primarily through anti-inflammatory mechanisms rather than direct enhancement of endogenous antioxidant defenses.

Collectively, quercetin demonstrates synergistic activity against colorectal cancer through concurrent suppression of inflammatory mediators (TNF-α→COX-2/iNOS axis) and oxidative effectors (LPO/NO/G6PD). TNF-α and iNOS emerge as pivotal nodes within this regulatory network. Future investigations should prioritize elucidating quercetin’s regulatory dynamics within the tumor microenvironment—particularly macrophage polarization—and its influence on metabolic reprogramming to further clarify pathway interdependencies.

### 5.2 Future directions and implications for clinical practice

This study explored the effects of quercetin on colorectal cancer cell proliferation, apoptosis, inflammatory response, and oxidative stress, suggesting its potential as a clinical alternative therapy, with positive implications for clinical research.

In our study, we identified associations between different substances in the pathogenesis of colorectal cancer, emphasizing the need to view these changes as a dynamic, interconnected process. For example, the inflammatory response interacts with the oxidative stress response. Therefore, further in-depth studies on the tumor microenvironment (TME) or gut microbiota are warranted. According to relevant studies, quercetin also plays a role in remodeling the tumor microenvironment and regulating the immune microenvironment. For instance, quercetin can suppress Wnt16 expression, thereby decreasing tumor cell resistance to chemotherapeutic agents and helping to remodel the tumor matrix (Hu et al., 2017; Zhao et al., 2024). By suppressing the autophagy of M2 type tumor-associated macrophages and triggering their conversion to M1 type, the proliferation of cancer cells is inhibited (Chen et al., 2014). Quercetin decreases neutrophil infiltration in tumors and shifts tumor-associated neutrophils from the tumor-promoting N2 type to the anti-tumor N1 type (Fang et al., 2024; Shaul et al., 2016). However, there is no general consensus on the role of quercetin in the colorectal cancer microenvironment and the specific relationship between the tumor microenvironment of colorectal cancer and inflammatory response and oxidative stress. Therefore, further study on the mechanism and regulatory pathways of quercetin in CRC tumor microenvironment will help to develop new therapeutic strategies and improve the therapeutic effect of CRC.

Additionally, contrary to popular belief, quercetin, as a natural flavonoid, has a favorable safety profile and is considered liver- and kidney-friendly (Chen et al., 2022). However, it remains unclear whether long-term use of quercetin could trigger resistance or pose safety concerns (Andres et al., 2018). Furthermore, during the literature screening, we excluded animal experiments on the combination of quercetin with other drugs, and thus could not draw conclusions on whether such combinations would affect efficacy or cause toxic side effects. It is also unknown whether there are other substances that enhance the possibility of quercetin treatment when combined. Therefore, future studies are needed to investigate the long-term efficacy and potential drug combinations involving quercetin in the treatment of CRC.

### 5.3 Limitations

This meta-analysis has elucidated some mechanisms of quercetin in colorectal cancer treatment, but significant limitations persist. The 17 included animal studies showed marked differences in key aspects, causing I^2^ values to frequently reach 75%–97% (ACF analysis I^2^ = 85%, tumor incidence I^2^ = 82%). Such high heterogeneity implies that the studies may not have measured the same true effect, making the pooled standardized mean difference (SMD) more of a weighted average than a generalizable biological constant. The following four types of differences are the main drivers of heterogeneity and further weaken the ability to draw definitive conclusions.

#### 5.3.1 Diversity in animal models and carcinogens

The methods to induce colorectal cancer in the control groups varied, including AOM, DMH, MNU, AOM+DSS, and blank controls. These different methods lead to distinct molecular pathological features. For example, AOM/DSS models mainly drive colorectal cancer development through inflammation, while DMH directly induces mutations via genotoxicity. Such differences may cause inconsistent effects of quercetin across models. Additionally, variations in animal strains (Sprague-Dawley, Wistar, BALB/c, and ICR mice) and gender can also lead to potential differences. Experimental environmental factors such as light cycle, temperature, and antibiotic use can all alter the gut microbiota, thereby affecting quercetin’s bioconversion and study outcomes.

#### 5.3.2 Heterogeneity in intervention protocols

The dose and administration route of quercetin in the experimental groups showed wide variations. Doses ranged from 50 to 200 mg/kg, and administration methods included oral gavage, intraperitoneal injection, and dietary incorporation. Differences in bioavailability can lead to fluctuations in drug concentration in the blood, affecting the consistency of therapeutic effects. Various administration routes significantly impact the drug’s Cmax, Tmax, and first-pass effect, potentially causing large differences in the concentration of free quercetin in target tissues. These factors collectively affect the nonlinear relationship between dose and effect, greatly reducing the biological significance of the “average SMD” while still retaining some meaning. The study periods in the included studies also varied from 4 to 24 weeks, which may lead to inadequate representation of changes in outcome indicators. Short-term studies may only capture early reductions in ACF and fail to reflect the cumulative effects of quercetin on chronic pathological processes. Longer-term studies are needed to observe the progression from adenoma to colorectal cancer. Ignoring the study period in summaries may result in incorrect overall effect assessments.

#### 5.3.3 Differences in data standardization and endpoint measurement timing

Differences in reporting standards may exist. For example, some studies reported β-catenin expression as a relative quantity (target protein to loading control ratio), while others used absolute concentrations, leading to significant discrepancies. Variations in sampling time points can also cause inconsistent study results. Some studies administered quercetin immediately after inducing colorectal cancer, while others initiated intervention only after tumor formation, potentially leading to misalignment in the evaluation of quercetin’s efficacy. Additionally, differences in the timing of euthanasia after the last quercetin administration can alter the values of outcome indicators, possibly due to dynamic rebounds in tissue regeneration and apoptosis signals.

In summary, the current pooled estimates have limitations in reflecting the intrinsic effects of quercetin due to the high heterogeneity across studies. Under such extreme heterogeneity, pooled effect sizes are prone to the influence of extreme study weights, and funnel plots, trim-and-fill methods, and Leave-one-out sensitivity analyses may not fully correct these biases. Future research should focus on conducting well-designed, large-sample animal studies and exploring the dynamic regulatory mechanisms of quercetin within the tumor microenvironment to promote its clinical translation.

## 6 Conclusion

A meta-analysis was carried out to evaluate the effects of quercetin on colorectal cancer in animal studies. This study verified the potential of quercetin as a target drug for CRC and provided an evaluation based on data analysis. This paper suggests that quercetin significantly reduces tumor incidence and the number of abnormal crypt lesions, and has notable effects on cell proliferation, apoptosis, inflammatory response, and oxidative stress. This information could aid in the study of quercetin dosing regimens or treatment strategies in clinical practice. However, to accurately assess quercetin’s role in CRC treatment, as well as potential drug combinations and side effects in clinical practice, our results need to be verified through human experiments and clinical trials.

## Data Availability

The original contributions presented in the study are included in the article/supplementary material, further inquiries can be directed to the corresponding author.

## Abbreviations

5.FU, 5-Fluorouracil; 95% CI, 95% confidence intervals; CRC, Colorectal Cancer; VEGF, vascular endothelial growth factor; EGFR, epidermal growth factor receptor; AOM, Azoxymethan; DSS, 1,2-dimethylhydrazine; MNU, N-Nitroso-N-methylurea; DSS, Dextran Sulfate Sodium Salt; ACF, aberrant crypt foci; PCNA, Proliferating Cell Nuclear Antigen; Bcl-2, B-cell lymphoma-2; TNF-α, tumor necrosis factor-α; IL-1β, Interleukin-1β; iNOS, inducible nitric oxide synthase; COX-2, cyclooxygenase-2; GSH, glutathione; GST, glutathione S-transferase; LPO, lipid peroxidation; SOD, Superoxide dismutase; CAT, Catalase; CEA, carcinoembryonic antigen; G6PD, glucose-6-phosphate dehydrogenase; NO, Nitric Oxide.
